# *BigR* is a sulfide sensor that regulates a sulfur transferase/dioxygenase required for aerobic respiration of plant bacteria under sulfide stress

**DOI:** 10.1038/s41598-018-21974-x

**Published:** 2018-02-22

**Authors:** Nayara Patricia Vieira de Lira, Bianca Alves Pauletti, Ana Carolina Marques, Carlos Alberto Perez, Raquel Caserta, Alessandra Alves de Souza, Aníbal Eugênio Vercesi, Adriana Franco Paes Leme, Celso Eduardo Benedetti

**Affiliations:** 1 Brazilian Biosciences National Laboratory (LNBio), Brazilian Center for Research in Energy and Materials (CNPEM), CEP 13083-100 Campinas, SP Brazil; 2 Department of Clinical Pathology, Faculty of Medical Sciences, State University of Campinas, 13083-887 Campinas, SP Brazil; 3 Brazilian Synchrotron Light Laboratory (LNLS), Brazilian Center for Research in Energy and Materials (CNPEM), CEP 13083-100 Campinas, SP Brazil; 4 Agronomic Institute of Campinas, Citriculture Research Center ‘Sylvio Moreira’, CEP 13490-970 Cordeirópolis, SP Brazil

## Abstract

To cope with toxic levels of H_2_S, the plant pathogens *Xylella fastidiosa* and *Agrobacterium tumefaciens* employ the *bigR* operon to oxidize H_2_S into sulfite. The *bigR* operon is regulated by the transcriptional repressor *BigR* and it encodes a bifunctional sulfur transferase (ST) and sulfur dioxygenase (SDO) enzyme, *Blh*, required for H_2_S oxidation and bacterial growth under hypoxia. However, how *Blh* operates to enhance bacterial survival under hypoxia and how *BigR* is deactivated to derepress operon transcription is unknown. Here, we show that the ST and SDO activities of *Blh* are *in vitro* coupled and necessary to oxidize sulfide into sulfite, and that *Blh* is critical to maintain the oxygen flux during *A. tumefaciens* respiration when oxygen becomes limited to cells. We also show that H_2_S and polysulfides inactivate *BigR* leading to operon transcription. Moreover, we show that sulfite, which is produced by *Blh* in the ST and SDO reactions, is toxic to *Citrus sinensis* and that *X. fastidiosa*-infected plants accumulate sulfite and higher transcript levels of sulfite detoxification enzymes, suggesting that they are under sulfite stress. These results indicate that *BigR* acts as a sulfide sensor in the H_2_S oxidation mechanism that allows pathogens to colonize plant tissues where oxygen is a limiting factor.

## Introduction

Hydrogen sulfide (H_2_S) is a gaseous molecule that is well known for its strong odor and toxicity. In the past few years, however, H_2_S has gained the status of a biological effector molecule in higher organisms, since it is produced in a regulated manner by many cell types and acts as a signaling molecule in a variety of physiological processes, both in mammals and plants^1–3^.

Microorganisms, including bacteria, also produce H_2_S^4^. Sulfur-reducing bacteria, for instance, can reduce sulfur compounds into H_2_S to produce energy under anaerobic conditions^5^. Other bacterial species, on the other hand, can also generate H_2_S from the protein catabolism and degradation of organic matter^6^.

Despite its beneficial role in preventing oxidative stress, H_2_S can become toxic to both mitochondria and bacterial cells, primarily because it blocks aerobic respiration through inhibition of the cytochrome c oxidase^7–9^. To circumvent such problem, bacterial cells, plant and animal mitochondria have evolved common mechanisms to eliminate H_2_S^10–16^.

In both plant and animal mitochondria, as well as in some bacterial species, three enzymes, including the Sulfide Quinone Oxidoreductase (SQOR), Thiosulfate Sulfur Transferase (TST) and Sulfur Dioxygenase (SDO) catalyze the oxidization of H_2_S into sulfite^10–12,14–16^. In the first enzymatic reaction catalyzed by SQOR, H_2_S is complexed with sulfite to form thiosulfate. The sulfane sulfur of thiosulfate is subsequently transferred to reduced glutathione (GSH) by the action of TST to form glutathione persulfide (GSSH), which is then oxidized into sulfite by the action of SDO, regenerating GSH. In human mitochondria, the malfunctioning of SDO, known as ETHE1, is the cause of Ethylmalonic Encephalopathy syndrome^12,17^. Because sulfite is also toxic to cells, animal mitochondria further oxidizes it into sulfate, whereas in plant pathogenic bacteria such as *Xylella fastidiosa* and *Agrobacterium tumefaciens*, sulfite is secreted to the medium by a sulfite exporter^13,14,18,19^.

We have shown that this mechanism of H_2_S detoxification in *X. fastidiosa* and *A. tumefaciens*, the causal agents of the Citrus Variegated Chlorosis (CVC) and Crown Gall disease, respectively, is encoded by the *bigR* operon. The *bigR* operon is regulated by *BigR* (biofilm growth-associated repressor), a winged helix-turn-helix repressor that recognizes the −10 region of the operon, blocking its transcription^13,20^. The repressor role of *BigR* is governed by the redox status of two conserved cysteines (Cys42 and Cys108) which, when oxidized into a disulfide bond, alter the conformation of the winged-helix DNA-binding region of the repressor, leading to repressor-DNA dissociation and operon activation^13^.

In addition to *BigR*, the *bigR* operon encodes a two-domain DUF442-ETHE1 protein, originally named *Blh* (β-lactamase-like hydrolase), and a TauE-related sulfite transporter^13,20^. Protein sequence alignments and structural modeling studies suggested that the *Blh* DUF442 domain might function as a rhodanese or sulfur transferase (ST), whereas its β-lactamase domain would function as an SDO similar to human ETHE1^13^. In fact, genetic studies showed that *Blh* becomes critical to bacterial growth under hypoxia or under conditions where H_2_S is produced^13^. This is particularly relevant for aerobic organisms like *X. fastidiosa* and *A. tumefaciens* which colonize plant tissues where the O_2_ tension is low. In such environment, H_2_S accumulation could potentially inhibit bacterial respiration. Although the lack of *Blh* impairs bacterial growth under oxygen-limited conditions^13^, how exactly *Blh* enhances bacterial survival under hypoxia or H_2_S stress was unclear. In addition, how *BigR* is deactivated to derepress operon transcription was also unknown^13^.

Here we show that the DUF442 and ETHE1 domains of *Blh* display ST and SDO activities upon GSH and GSSH, respectively, producing sulfite, and that such enzymatic activities are *in vitro* coupled and required to maintain the O_2_ flux during bacterial respiration when O_2_ becomes limited to cells. Our data also reveal that H_2_S and polysulfides react with Cys108 and induce the Cys42-Cys108 disulfide bond formation in *BigR*, leading to repressor inactivation and operon transcription. We also present evidence suggesting that citrus plants infected with *X. fastidiosa* are under sulfite stress.

## Results

### The ETHE1 domain of *Blh* is an iron-containing sulfur dioxygenase

**Figure 1:**
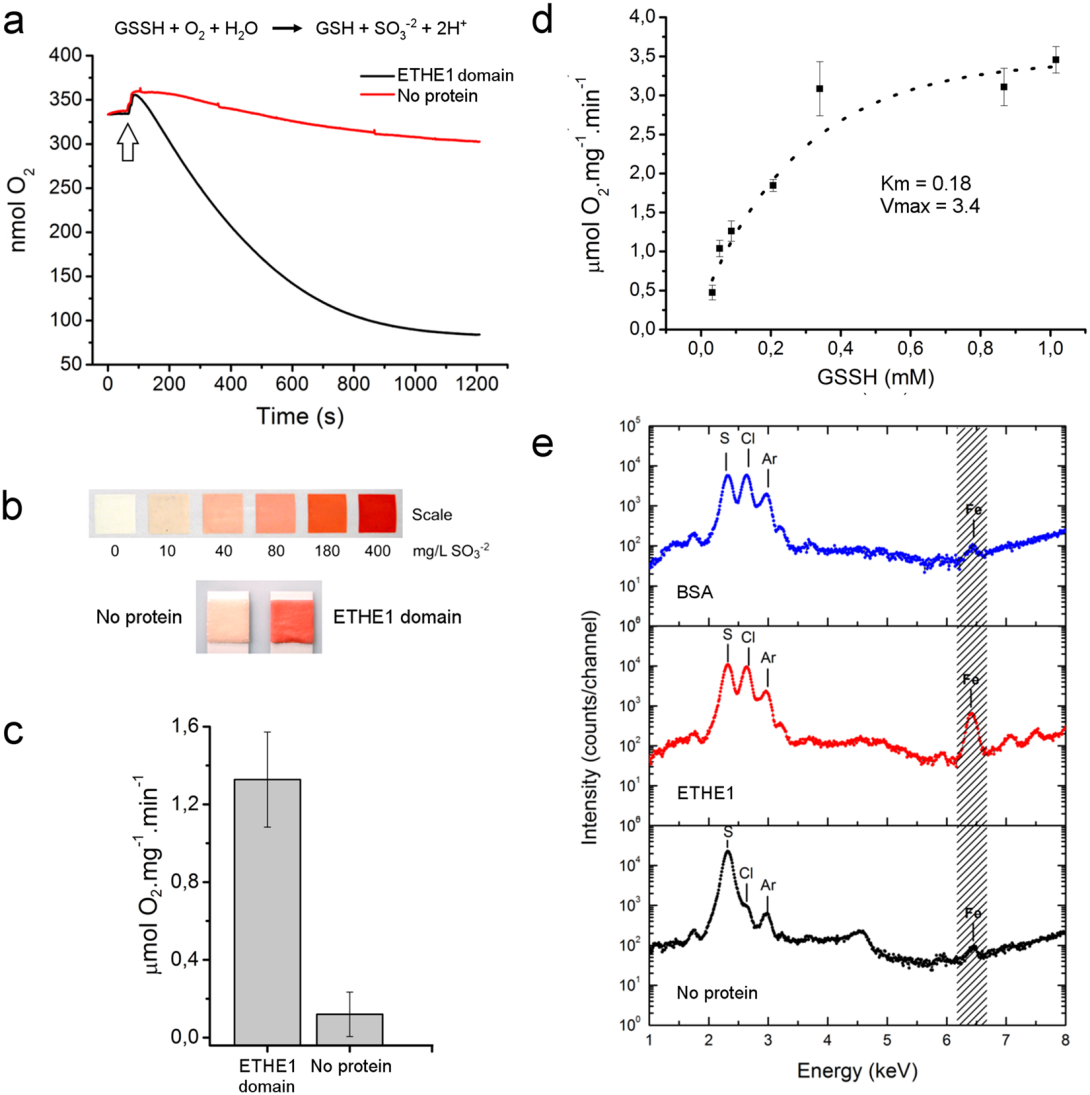
The ETHE1 domain of *Blh* is an iron-containing sulfur dioxygenase. **(a**) SDO activity of the *Blh* ETHE1 domain (black line), relative to buffer with no protein (red line), according to the reaction scheme shown. Oxygen consumption, as a measure of the SDO activity of the *Blh* ETHE1 domain, occurs only after the addition of substrate GSSH to the reaction mixture (arrow). (**b**) Sulfite production at the terminus of the SDO reaction estimated by the sulfite test strip, according to the scale. (**c**) Average O_2_ concentration per mg of protein (ETHE1 domain) per min at 25 °C, relative to control (no protein in the reaction mixture). Values are the mean of three independent measurements and error bars indicate standard deviations. (**d**) SDO activity as function of the GSSH concentration, showing that the ETHE1 domain displays a Michaelis-Menten kinetics with an estimated apparent *Km* and *V*_*max*_ values around 0.18 mM GSSH and 3.4 µmol O_2_ mg^−1^ min^−1^, respectively. (**e**) X-ray fluorescence analysis showing higher levels of iron in the ETHE1 domain sample, relative to buffer alone (no protein) or BSA, as negative control samples.

In our previous work, we provided evidence that the ETHE1 domain of *Blh* would function as an SDO like the mitochondrial ETHE1 protein, since the *blh*-defective mutant of *A. tumefaciens* accumulated higher levels of H_2_S^13^. In addition, based on structural modeling studies, we hypothesized that the *Blh* ETHE1 domain would use GSSH as a substrate^13^. To verify these assumptions, the recombinant ETHE1 domain of *Xylella Blh*, corresponding to residues 143 to 407, was purified and tested for the SDO activity upon GSSH. In agreement with the predictions, we found that O_2_ is consumed in the presence of GSSH and the *Blh* ETHE1 domain, and that sulfite is generated as a reaction product (Fig. 1a and b). Typically, ~1.3 ± 0,3 µmol O_2_ was consumed per mg of the ETHE1 domain per min at 25 °C (Fig. 1c).

To better characterize the kinetic properties of the *Blh* ETHE1 domain, the SDO reaction was performed in the presence of increased amounts of GSSH. The results indicate that the ETHE1 domain shows an ordinary Michaelis-Menten kinetics with estimated apparent *Km* and *V*_*max*_ of approximately 0.18 mM GSSH and 3.4 µmol O_2_ mg^−1^ min^−1^, respectively (Fig. 1d). These kinetics parameters are comparable to those of other characterized SDOs^11,15^.

Our molecular modeling studies also suggested that a metal ion, possibly iron, could be part of the active site of the *Blh* ETHE1 domain^13^. Consistent with this, X-ray fluorescence analysis revealed higher levels of iron in the ETHE1 domain sample, relative to buffer alone or BSA (bovine serum albumin), used as negative control samples (Fig. 1e). This result is in line with the fact that iron was also found to bind the *Blh*-related *CstB* and human ETHE1 enzymes^15,21^.

### The *Blh* DUF442 domain is a sulfur transferase

**Figure 2:**
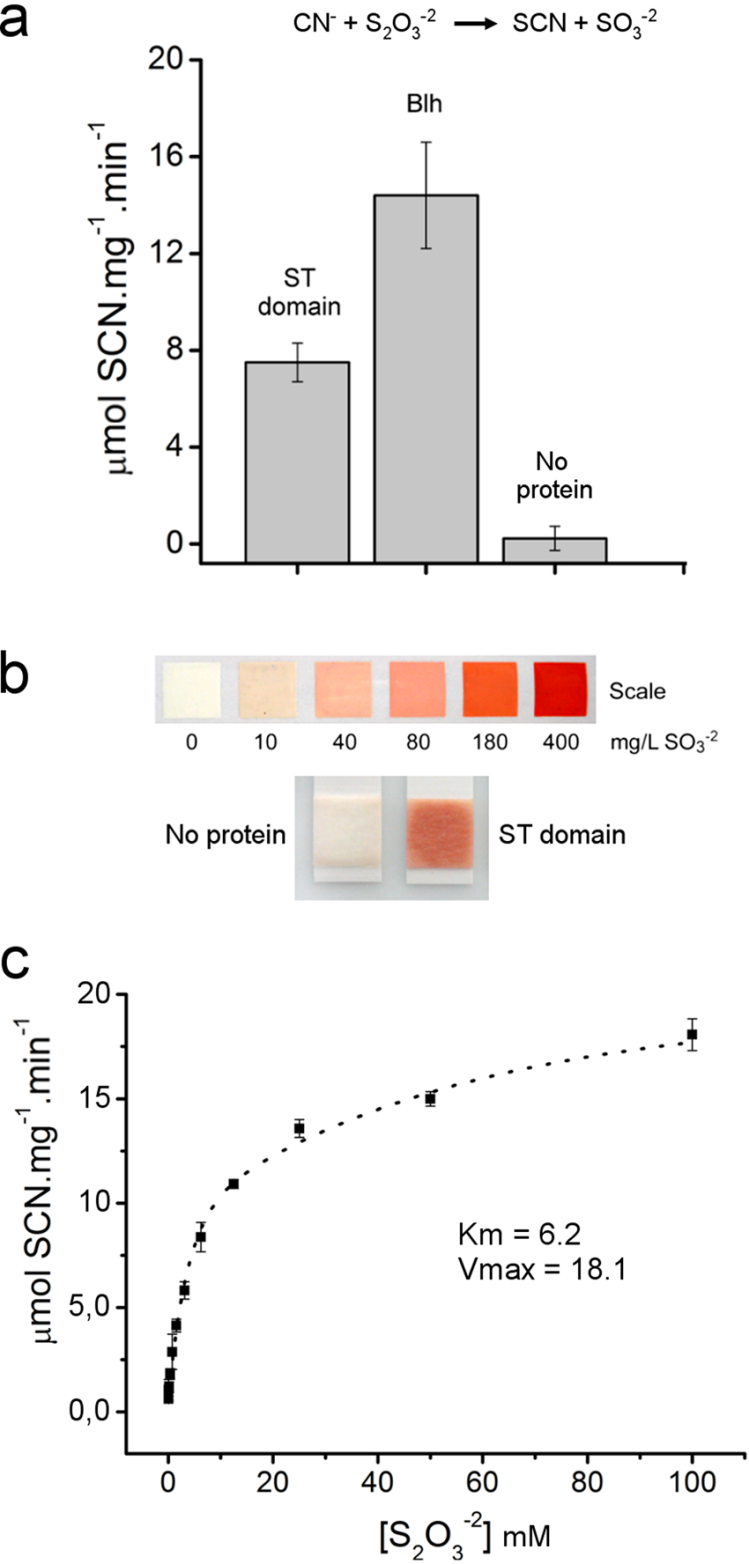
The *Blh* DUF442 domain is a sulfur transferase. (**a**) ST activity of the full-length *Blh* or its DUF442 domain alone (ST domain), relative to buffer with no protein. The ST activity, as a measure of iron-SCN formation, used thiosulfate as the sulfur donor and cyanide as the sulfur acceptor, according to the reaction scheme shown. Values are the mean of nine independent measurements and error bars indicate the standard deviation. (**b**) Sulfite production at the terminus of the ST reaction estimated by the sulfite test strip, according to the scale, indicating that both the full-length *Blh* and DUF442 domain catalyzed the transfer of the sulfane sulfur of thiosulfate to cyanide, generating SCN and sulfite as the reaction products. (**c**) ST activity of full-length *Blh* as function of the thiosulfate concentration, showing that *Blh* displays a Michaelis-Menten kinetics with an estimated apparent *Km* and *V*_*max*_ values of ~6.2 mM thiosulfate and 18.1 µmol SCN mg^−1^ min^−1^, respectively.

Protein sequence alignment and molecular modeling studies suggested that despite sharing the same fold of protein tyrosine phosphatases, the *Blh* DUF442 domain was not only structurally similar to the catalytic domain of the *E. coli* rhodanese YnjE^22^, but also presented a conserved rhodanese active site^13^. To further investigate this, the recombinant DUF442 domain of *Xylella Blh* (residues 1 to 141) was purified and tested for a rhodanese or sulfur transferase (ST) activity using thiosulfate as a sulfur donor and cyanide as a sulfur acceptor^23^. The results show that the DUF442 domain of *Blh* was sufficient to catalyze the transfer of the sulfane sulfur of thiosulfate to cyanide, generating thiocyanate (SCN) and sulfite as the reaction products (Fig. 2a and b). The recombinant full-length *Blh* also showed ST activity upon thiosulfate and cyanide which was, on average, twice of that exhibited by the DUF442 domain alone (Fig. 2a).

Because the DUF442 domain was relatively less soluble and active than the full-length *Blh* when expressed in *E. coli* cells, the kinetics properties of the DUF442 domain was determined using the full-length *Blh* protein. The apparent *Km* and *V*_*max*_ values for the *Blh* ST activity were 6.2 mM thiosulfate and 18.1 µmol SCN mg^−1^ min^−1^, respectively (Fig. 2c), which are also comparable to the *Km* and *V*_*max*_ values determined for the *S. aureus CstB*^15^. These data thus show that the DUF442 domain of *Blh* functions as a sulfur transferase and, therefore, will be referred here as the *Blh* ST domain.

### The ST and SDO activities of *Blh* are coupled

**Figure 3:**
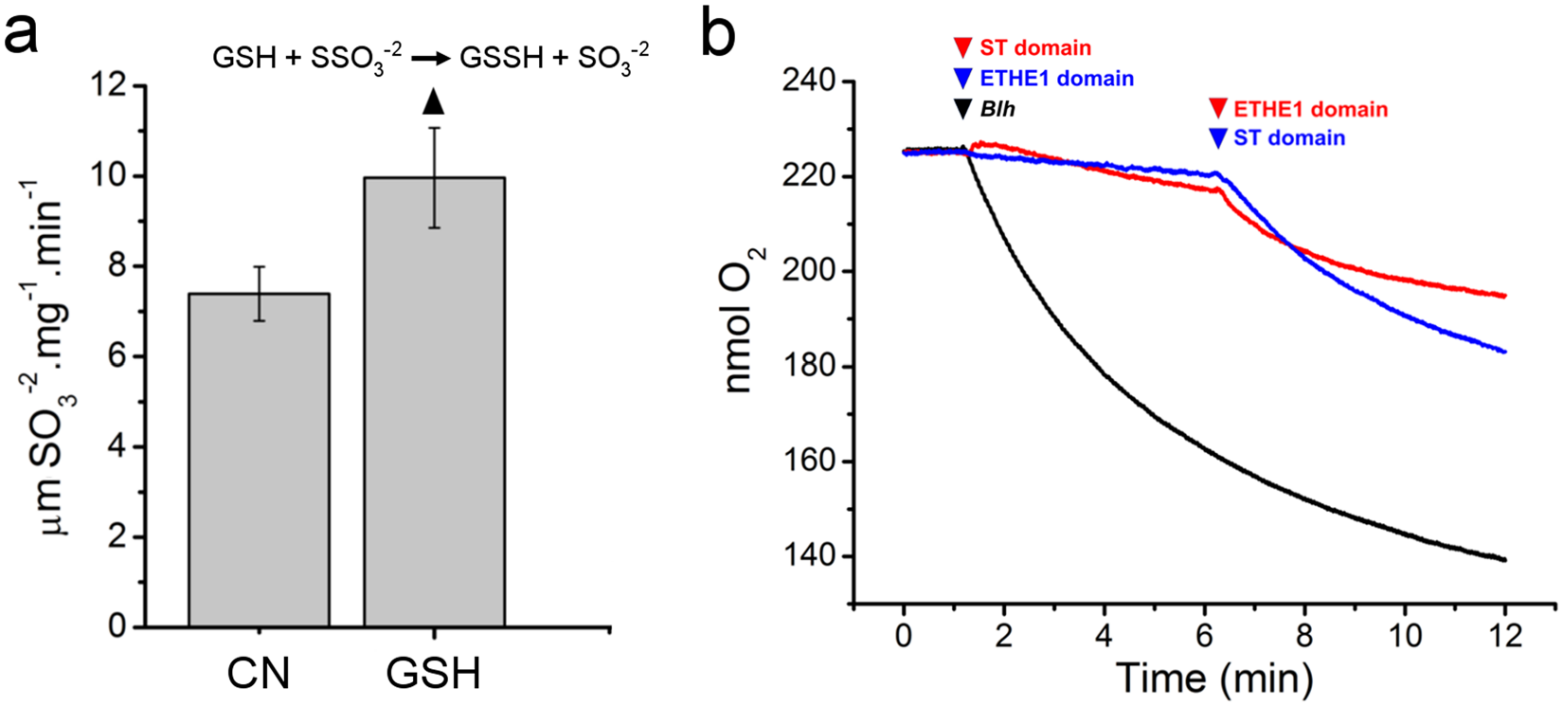
The couple ST and SDO activities of *Blh*. **(a)** ST activity of the *Blh* ST domain as a measure of sulfite production when cyanide (CN) or GSH are used as sulfur acceptors and thiosulfate as the sulfur donor. Values are the mean of three independent measurements and error bars indicate the standard deviation. In addition to sulfite, the ST activity upon GSH is expected to generate GSSH, according to the reaction scheme shown. (**b**) O_2_ consumption as a measure of the SDO activity of the *Blh* ETHE1 domain showing that the O_2_ concentration in the medium is not significantly altered when the ST domain is added to the reaction mixture containing thiosulfate and GSH. Nevertheless, O_2_ consumption increases significantly soon after the addition of the ETHE1 domain to the reaction mixture (red line). Similar results are observed when the ETHE1 domain is added first to the reaction mixture (blue line). When the full-length *Blh* is used, a sharper decrease in the O_2_ levels is observed (black line), indicating that the ST and SDO activities of *Blh* are *in vitro* coupled.

We found that the ST domain of *Blh* can also use GSH as a sulfur acceptor to produce sulfite and GSSH when thiosulfate is the sulfur donor (Fig. 3a). Considering that the *Blh* ETHE1 domain uses GSSH as substrate and its SDO activity generates GSH, we tested whether the ST and SDO activities of *Blh* were coupled. To verify this, the ST and ETHE1 domains of *Blh* were purified separately and O_2_ consumption, as a result of the SDO activity (Fig. 1a), was measured in the reaction. The results show that O_2_ consumption is not significantly altered when the ST or the ETHE1 domain is added alone into the reaction mixture containing thiosulfate and GSH. However, O_2_ consumption increases significantly as soon as the second *Blh* domain is added to the reaction mixture (Fig. 3b). When the full-length *Blh* is used, an instant and sharper O_2_ consumption is observed (Fig. 3b). Together, these results confirm that the ST and SDO activities of *Blh* are *in vitro* coupled and that GSH, consumed in the ST reaction, is restored in the SDO reaction.

### *Blh* is essential to maintain aerobic respiration under oxygen-limited conditions

We showed previously that the *bigR* operon is important for H_2_S detoxification and that *Blh* is required to sustain bacterial growth under O_2_-limited concentrations^13^. Given that H_2_S competes with O_2_ for cytochrome c oxidase binding, we decided to investigate the role of *Blh* as a sulfide oxidation enzyme, particularly when O_2_ becomes limited to the cells. To test this, we performed respiration assays using the wild type and corresponding *blh*^*−*^ and *bigR*^−^ deletion mutants of *A. tumefaciens* described previously^20^. The bacterial cells were incubated in medium containing glucose and O_2_ consumption was monitored until its full depletion.

**Figure 4:**
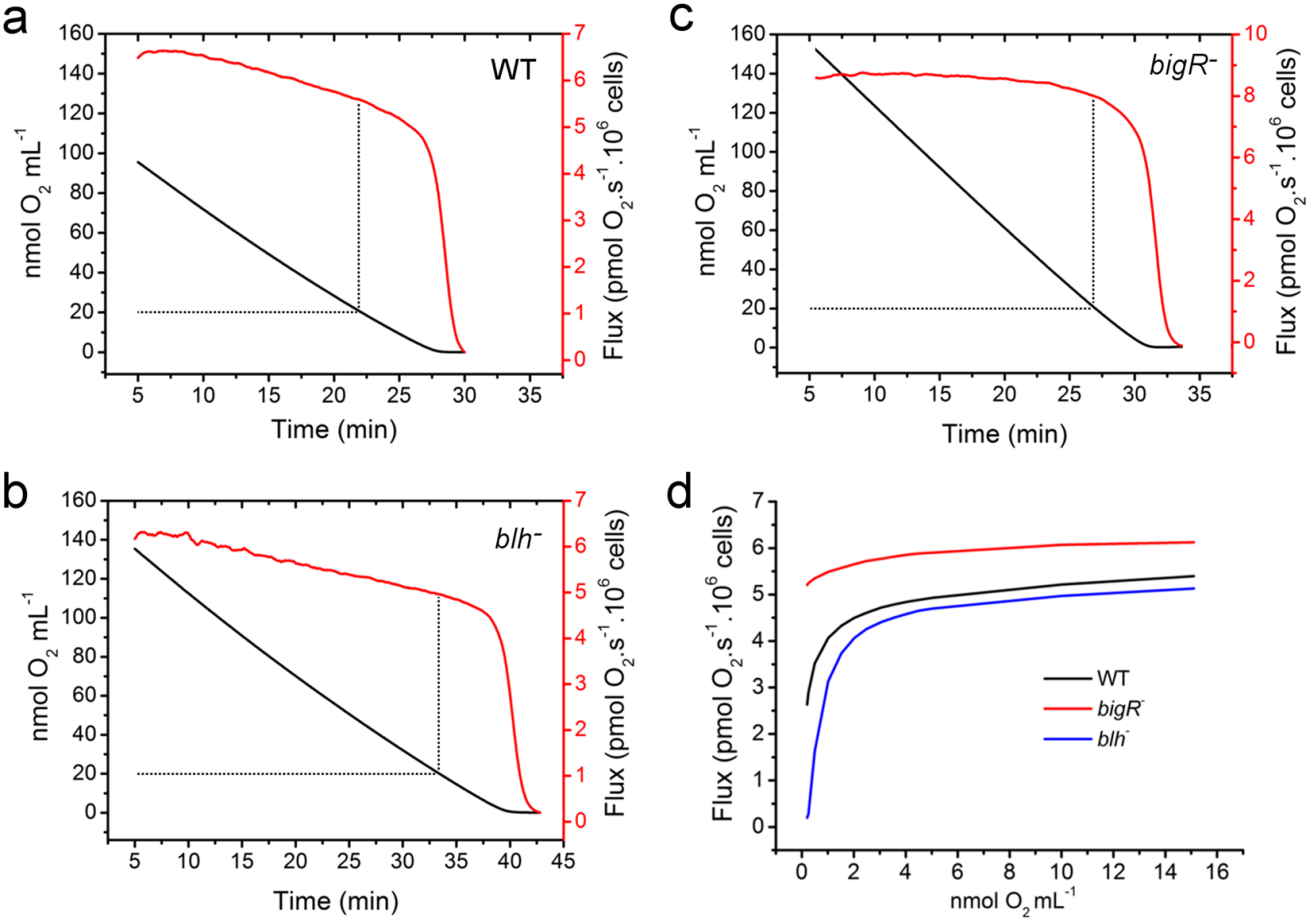
*Blh* is required to maintain aerobic respiration under O_2_-limited conditions. O_2_ consumption (black lines) and O_2_ flux (red lines), as a measure of aerobic respiration, exhibited by the wild type (**a**) *A. tumefaciens* and corresponding *blh*^−^ (**b**) and *bigR*^−^ (**c**) cells in medium containing glucose. The *blh*^*−*^ cells showed a lower O_2_ flux (**b**) compared to the wild type cells (**a**) under O_2_-replete conditions (above 20 nmol mL^−1^), whereas the *bigR*^*−*^ cells (**c**) showed a higher O_2_ flux relative to the wild type cells. (**d)** O_2_ flux as a function of the O_2_ concentration showing that the differences in the O_2_ flux between the *blh*^*−*^ and *bigR*^*−*^ mutants, relative to the wild type cells, increased significantly as the O_2_ concentration in the medium approaches zero. The O_2_ flux of *blh*^*−*^ cells at 0.2 nmol mL^−1^ O_2_ was approximately 10 × lower than that of the wild type cells, but nearly the same of that of the wild type cells at 5.0 nmol mL^−1^ O_2_. Conversely, the *bigR*^*−*^ cells maintained a high O_2_ flux even at 0.2 nmol mL^−1^ O_2_, compared to the wild type and *blh*^*−*^ cells.

The O_2_ flux, as a measure of the aerobic respiration, was in the order of 10^−18^ mol cell^−1^s^−1^ for the three bacteria, which is consistent with the average of O_2_ utilization described for other cell types^24^. We found that the *blh*^*−*^ mutant showed a relatively lower O_2_ flux compared to the wild type cells under O_2_-replete conditions (above 20 nmol mL^−1^), whereas the *bigR*^*−*^ mutant, on the other hand, a slightly higher O_2_ flux relative to the wild type cells (Fig. 4a, b and c). Interestingly however, the differences in the O_2_ flux between the *blh*^*−*^ and *bigR*^*−*^ mutants, relative to the wild type cells, increased significantly as the O_2_ concentration in the medium approached zero (Fig. 4d). The O_2_ flux of *blh*^*−*^ cells, for instance, was approximately one tenth of that of the wild type cells at 0.2 nmol mL^−1^ O_2_, but nearly the same of that of the wild type cells at 5.0 nmol mL^−1^ O_2_ (Fig. 4d). On the other hand, the *bigR*^*−*^ cells maintained a higher O_2_ flux compared to the wild type and *blh*^*−*^ cells even at 0.2 nmol O_2_ mL^−1^ (Fig. 4d). These results thus confirm that O_2_ consumption is affected in the *blh*^*−*^ mutant and that the *bigR* operon is critical to improve aerobic respiration under hypoxia, a condition experienced by *Xylella* and *Agrobacterium* cells inside plant tissues.

### Sulfite is toxic to citrus cells and accumulates in CVC plants

**Figure 5:**
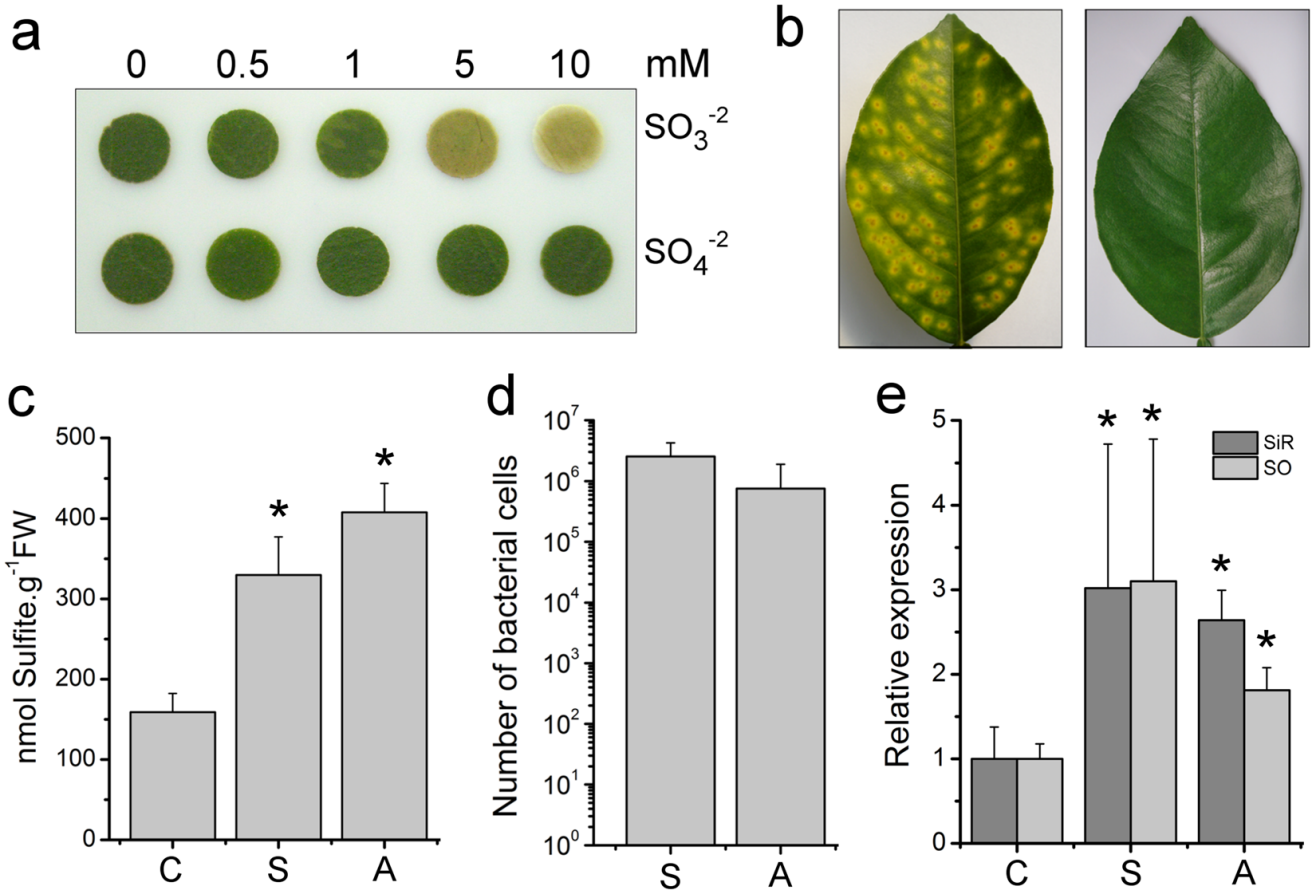
*Xylella*-infected plants are under sulfite stress. **(a**) Leaf discs of Pineapple sweet orange incubated with increased amounts of sulfite or sulfate, showing that sulfite, but not sulfate, induces chlorosis in citrus. (**b**) Example of a symptomatic CVC leaf of Pineapple plants showing typical CVC lesions surrounded by chlorotic halos (left panel), compared with an asymptomatic leaf (right panel). (**c**) Sulfite levels measured in symptomatic (S) and asymptomatic (A) CVC leaves showing higher sulfite levels in symptomatic and asymptomatic leaves relative to non-infected control (C) plants. Values are the means of six independent measurements and error bars indicate the standard deviation, whereas asterisks indicate statistically significant difference between the means. (**d**) Quantitative PCR analysis showing high bacterial titles in both symptomatic and asymptomatic leaves of CVC plants. No PCR amplification is detected in non-infected plants. (**e**) Quantitative PCR analysis showing the relative mRNA levels of SiR and SO between symptomatic and asymptomatic CVC leaves, compared to non-infected control plants. Values in ‘d’ and ‘e’ are the means of five independent biological replicates and asterisks indicate statistically significant difference between the means.

The results presented in Figs 1b, 2b and 3a show that sulfite is generated by both ST and SDO activities of *Blh*. In contrast to mitochondria, where sulfite produced by the TST and ETHE1 pathway is further oxidized to non-toxic sulfate before it is secreted^14^, in *A. tumefaciens*, sulfite is excreted to the medium^13^. Because sulfite is toxic to plant cells^25–28^, we suspected that sulfite secreted by the action of the *bigR* operon could become toxic to the host plant, as bacterial colonization progresses. To explore this idea, we incubated sweet orange leaf discs in the presence of increased amounts of sulfite or sulfate. We found that sulfite, but not sulfate, caused a dose-dependent chlorosis in citrus tissues, indicating that it is toxic to citrus cells (Fig. 5a). Because sulfite caused the yellowing of the citrus leaf discs, we hypothesized that it might contribute to the CVC symptoms, since typical CVC lesions are surrounded by chlorotic halos (Fig. 5b). To investigate this, we measured the sulfite levels in symptomatic and asymptomatic CVC plants, relative to non-infected (healthy) plants. Sulfite levels in non-infected citrus leaves were in the range of 150 nmol per gram of fresh weigh (Fig. 5c), which is close to what has been reported for tomato and Arabidopsis^27,28^. We found that the sulfite levels were significantly higher in both symptomatic and asymptomatic leaves, relative to non-infected plants (Fig. 5c). Consistent with these results, bacterial quantification in the petioles of the corresponding leaves revealed high bacterial titles in both symptomatic and asymptomatic leaves (Fig. 5d).

To further investigate whether *Xylella*-infected leaves were under sulfite stress, the mRNA levels for Sulfite Reductase (SiR) and Sulfite Oxidase (SO), the principal enzymes responsible for sulfite detoxification in plants^26–28^, were examined. We found that both SiR and SO are significantly increased in leaves infected with *Xylella*, compared to non-infected leaves (Fig. 5e). Thus, although a clear link between sulfite levels and visible CVC symptoms could not be established, the results show a correlation between increased sulfite levels and bacterial cell population with the expression of sulfite detoxification genes in *Xylella*-infected plants, indicating that plants with CVC are under sulfite stress.

### H_2_S and polysulfides oxidize *BigR* and activate the *bigR* operon

**Figure 6:**
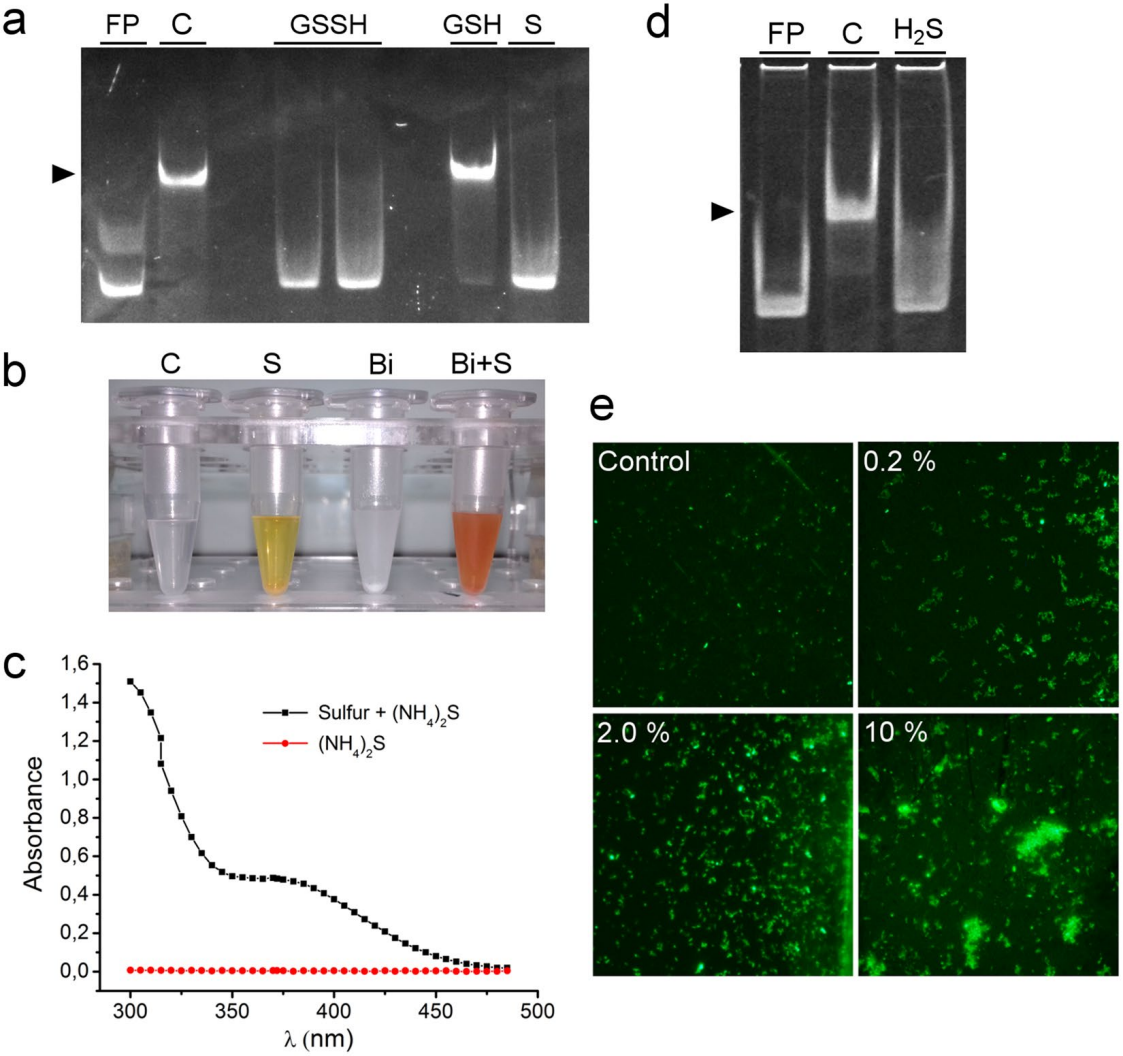
H_2_S and polysulfide oxidizes *BigR* and activates the *bigR* operon. **(a**) Gel-shift assay stained with ethidium bromide showing formation of the *BigR*-DNA complex (arrow) in the control mix reaction (C), or in the presence of GSH. The *BigR*-DNA complex is not only abolished in the presence of GSSH, but also in the presence of acetonic sulfur (S), used in combination with GSH to generate GSSH. FP designates the free probe. (**b**) Detection of HS^*−*^ and polysulfides in the commercial source of elemental sulfur. Acetone adjusted to pH 12 with potassium hydroxide was used as a control solution (C). Addition of sulfur (S) caused the solution to turn yellow indicating the presence of polysulfides in the sample. Addition of bismuth citrate (Bi) to the acetone solution formed a white precipitate which turned into an orange-brownish color with sulfur addition (Bi + S), indicating the presence of HS^*−*^ in the sample. (**c**) Absorbance curve of the ammonium sulfide solution (60 mM) in the presence and absence of sulfur showing peaks at 300 and 375 nm characteristic of polysulfides. (**d**) Gel-shift assay showing that the *BigR* protein treated with H_2_S gas no longer binds DNA. (**e**) Fluorescence images of *A. tumefaciens* cells carrying the GFP reporter plasmid for the *bigR* operon growing in the presence and absence of the polysulfide solution at 0.2%, 2.0% or 10% v/v. Cells exposed to the polysulfide solution show increased GFP fluorescence in a dose-dependent manner, indicating that HS^−^ and/or polysulfides are responsible for the operon activation.

*BigR* has been suggested to function as a redox sensor in the sense that it loses affinity to DNA, allowing transcription of the operon, when its Cys42 and Cys108 residues form an intra-chain disulfide bond^13^. To investigate what molecules might induce *BigR* oxidation, we performed gel-shift assays in the presence of GSSH, since GSSH and other persulfides were recently shown to regulate the *bigR*-related *cst* operon in *S. aureus* and the sulfide-responsive regulator *SqrR* in *Rhodobacter capsulatus*^15,29–31^. Notably, we found that although GSSH abolished the *BigR* binding to its target DNA, the control reaction containing only the elemental sulfur also disrupted the *BigR*-DNA complex (Fig. 6a). This result strongly suggested that sulfide or polysulfides, reported to be present in sulfur-containing compounds, was responsible for *BigR* oxidization. Indeed, we found that the elemental sulfur source used in our experiments contained polysulfides, as revealed by the characteristic yellow pigmentation in alkaline solution^32^ (Fig. 6b). The presence of polysulfides in the elemental sulfur source was also evidenced by the absorbance curve with maximum peaks at 300 and 375 nm^33^ (Fig. 6c). Moreover, our elemental sulfur source also reacted with bismuth ion at alkaline pH forming a brownish-orange pigment, a strong indication of the presence of H_2_S or the hydrosulfide ion (HS^−^) in solution (Fig. 6b). These results, together with the fact that polysulfides have recently been shown to link H_2_S to protein thiol oxidization^33^, led us to investigate whether *BigR* could directly sense the H_2_S gas. To test this, recombinant *BigR* was incubated with gaseous H_2_S in a stainless-steel jar for 30 min, and the protein was subjected to gel-shift assays. We found that *BigR* treated with H_2_S no longer bound the target DNA (Fig. 6d), indicating that H_2_S itself can lead to *BigR* Cys42-Cys108 disulfide bond formation and operon activation.

Because gaseous H_2_S could not be employed to treat bacterial cells in ordinary bacterial growth chambers, we instead tested whether our sulfur preparations containing HS^−^ and/or polysulfides could activate the *bigR* operon. Thus, *A. tumefaciens* cells carrying a GFP reporter gene driven by the *bigR* operon promoter^20^ were grown in the presence or absence of HS^−^/polysulfides. We found that the reporter cells exposed to the HS^−^/polysulfide solution showed increased GFP fluorescence in a dose-dependent manner (Fig. 6e), indicating that HS^−^ and/or polysulfides were responsible for the operon activation, and like the H_2_S gas, were inducing the disulfide bond formation in *BigR*. To confirm this, recombinant *BigR* that had been incubated with gaseous H_2_S or the HS^−^/polysulfide solution was subject to mass spectrometry analysis. The results show that treatments of *BigR* with the H_2_S gas or HS^−^/polysulfide solution led to the incorporation of a ‘SH’ or ‘SSH’ group to the sulfur atom of Cys108, forming cysteine persulfides (Supplementary Table S1). Moreover, the peptide that shows the disulfide bond involving Cys42 and Cys108^13^ was also detected in *BigR* samples treated with gaseous H_2_S (Supplementary Fig. S1). Thus, considering that cysteine persulfides can serve as intermediates of disulfide bond formation when a second cysteine is nearby^30,33^, the results show that *BigR* senses sulfide species and that H_2_S itself is the actual modulator of *BigR in vivo*.

### *BigR* is modified by sulfenic acid and glutathionylation at regulatory Cys residues

Sulfenic acid at cysteine residues has been reported to serve as an intermediate in disulfide bond formation^34–36^. In addition, H_2_S has been shown to react with sulfenic acid to form persulfides^37^. In the *BigR*-related transcriptional regulator *HlyU* from *Vibrio cholera*, Cys38 is modified to cysteine sulfenic acid^38^. Because this cysteine is structurally equivalent to Cys42 in *BigR*, we tested whether Cys42 in *BigR* could also be modified to cysteinyl sulfenic acid. Using dimedone, which specifically reacts with sulfenic acid^36^, we found that not only Cys42 but also Cys108 is modified to cysteine sulfenic acid (Table S1). Moreover, we found that Cys42 and Cys108 can also be linked to GSH (Table S1). Since GSH can react with cysteinyl sulfenic acid^34^, our results indicate that Cys42 and Cys108 in *BigR* can be modified to cysteinyl sulfenic acid to subsequently react with GSH or SH^−^.

## Discussion

In our previous work, we showed that the *bigR* operon was required for H_2_S detoxification to allow bacterial growth under O_2_-limited conditions and suggested that *Blh* was implicated in the oxidization of H_2_S into sulfite^13^. Moreover, we predicted that the DUF442 and ETHE1 domains of *Blh* would display independent but coupled enzymatic activities and that DUF442 was structurally related to bacterial rhodaneses^13^. Here, we confirmed that the SDO and ST/rhodanese activities of the ETHE1 and DUF442 domains of *Blh* are *in vitro* coupled, corroborating data that show that such H_2_S oxidation mechanism is conserved in bacteria, plant and animal mitochondria^10–12,14–16^.

Our data also unveiled the fundamental question of why *Blh* is critical for bacterial growth under hypoxia. Since H_2_S blocks aerobic respiration by competing with O_2_ for cytochrome C oxidase binding, *Blh* helps to maintain a high O_2_ flux, especially when O_2_ becomes a limiting factor to the cells. Importantly, a comparative genomics study indicated that strains of *X. fastidiosa* possess a simple respiratory complex with just one terminal oxidase that would allow aerobic respiration only at high O_2_ levels, as they seem to lack cytochromes with high O_2_ affinity^39^. Thus, for aerobic organisms like *X. fastidiosa* and *A. tumefaciens*, the *bigR* operon becomes essential for survival, since it allows these pathogens to respire inside xylem vessels and roots where the O_2_ concentration can be very low^40–42^.

Because sulfite is produced by the ST and SDO reactions catalyzed by *Blh*, and is toxic to plant cells^26–28^, we decided to investigate whether sulfite could contribute to CVC symptoms, since it also induced chlorosis in citrus leaves (Fig. 5a). Although there was no apparent direct correlation between increased sulfite levels and typical chlorosis in CVC symptomatic leaves (Fig. 5), we found a correlation between increased sulfite levels and *Xylella* population with the augmented expression of Sir and SO. Thus, although further studies will still be necessary to demonstrate whether sulfite secreted by *Xyllela* cells would play an important role in the development of CVC symptoms, our data indicate that plants infected with *Xylella* are under sulfite stress.

Another fundamental question that remained unanswered was regarding the molecule responsible for *BigR* oxidation and operon activation *in vivo*. Due to its redox potential, H_2_S was initially not considered as a candidate^13^. However, in light of the work by Greiner and colleagues^33^ who showed that, in spite of its redox potential, H_2_S can lead to protein disulfide bond formation via polysulfide intermediates, we decided to investigate whether polysulfides or H_2_S could oxidize *BigR* and activate the operon. Here, we show that solutions containing HS^−^ and/or polysulfides activated the *bigR* operon and induced persulfide formation at Cys108. Like the H_2_S-induced sulfhydration of human Protein Tyrosine Phosphatase PTP1B^43^, gaseous H_2_S flushed into solutions of *BigR* also induced sulfhydration of Cys108 and Cys42-Cys108 disulfide bond formation in the repressor. Notably, the sulfur atom of Cys108 is more surface exposed in the *BigR* 3D structure, compared with Cys42, and thus more prone to react with HS^−^ or polysulfides to form the cysteine persulfide (Supplementary Fig. S2a). Moreover, a comparison of the crystal structures of reduced (SH) and oxidized (S-S) *BigR* shows that the side chain of Trp104, which sits in between Cys42 and Cys108 in reduced *BigR* and prevents the disulfide bond formation, flips almost 90 degrees in the oxidized *BigR*^13^ (Fig. S2a and b). It is thus possible that the initial formation of a Cys108 persulfide in reduced *BigR* could induce a conformational change in the Trp104 side chain opening space for the two cysteines to interact (Supplementary Fig. S2b).

It is also noteworthy that Cys42 and Cys108 can undergo S-glutathionylation and S-sulfenylation. Even though these cysteines modifications were the result of *in vitro* reactions with recombinant *BigR*, it is known that cysteine sulfenic acid can react not only with GSH, but with H_2_S to form cysteine persulfides, which eventually lead to disulfide bond formation between vicinal cysteines^34,37,44,45^. Our data thus suggest that H_2_S/HS^−^ is likely to be the actual signaling molecule that activates the *bigR* operon via cysteine sulfhydration and disulfide or polysulfide bond formation in *BigR*. Therefore, instead of acting as a simple redox sensor, as previously suggested^13^, *BigR* appears to play a role as a H_2_S or sulfide sensor. In this regard, despite sharing similar features to the *S. aureus CstR* persulfide sensor^29,30^, *BigR* is phylogenetically related to the *SqrR* repressor recently shown to act as a sensor for H_2_S-derived reactive sulfur species^31^.

We hope that the data presented here indicating that *BigR* is a H_2_S sensor and that *Blh* is required for aerobic respiration under hypoxia will offer new opportunities for pathogen control.

## Methods

### Cloning, expression and purification of *Blh* and corresponding DUF442 and ETHE1 domains

The 9a5c *X. fastidiosa Blh* full-length gene (WP_010893291) and corresponding DUF442 and ETHE1 regions were amplified with the oligonucleotide pairs CATATGAGAATAGTAGACATC/CTCGAGTCACCATGCGGCACC, CATATGAGAATAGTAGACATC/GAGCTCGTTAGGCTTGACGTTCTAACCAGG and CATATGCAGACTCCGCGCGTTTCAGG/CTCGAGTCACCATGCGGCACC, respectively, and subcloned into the *Nde*I/*Xho*I-*Sac*I sites of pET28a vector for the expression of recombinant 6xHis-fusion proteins. The plasmids were verified by DNA sequencing and inserted into *Escherichia coli* BL21 pLySE cells, which were grown at 20 °C for 30 h in ZYM5052 medium^46^, supplemented with kanamycin (100 µg/mL), for protein production. The cells were suspended in lysis buffer (20 mM Tris-HCl, pH 7.4, 200 mM NaCl, 300 mM ammonium sulfate, 10% glycerol) and disrupted in a French press (25 kpsi) at 4 °C. The suspension was centrifuged and the recombinant proteins were purified on a NiNTA column (GE-Healthcare) equilibrated with lysis buffer. The column was washed with 10 column volumes of lysis buffer containing 50 mM imidazole and the proteins were eluted with lysis buffer containing 200 mM imidazole. Protein purity was verified by denaturing polyacrylamide gels and quantified on a NanoDrop. Protein yields were around 1.0, 0.6 and 4.0 mg/L for the full length *Blh*, ST and SDO domains, respectively.

### Enzyme assays

The SDO activity of the *Blh* ETHE1 domain was determined by measuring the O_2_ consumption in the reaction mixture, as described by Hildebrandt and Grieshaber^11^. The purified ETHE1 domain (12 µg) was incubated at 25 °C in 2 mL (final volume) of 100 mM potassium phosphate buffer, pH 7.4, containing 1 mM GSH. The reaction started with the addition of 30 µL of a saturated acetonic sulfur solution, and O_2_ consumption was measured on a Hansatech oxygraph. The sulfite concentration in the reaction mixture was estimated using the MERCKOQUANT sulfite test strips (Merck-Millipore, USA) at the end of the SDO reaction. For estimation of the specific SDO activity, *V*_*max*_ and *Km* values, the enzyme assays were performed with increasing amounts of GSSH (0 to 1.2 mM). The amount of GSSH obtained in the GSH/saturated acetonic sulfur solution was estimated by the methylene blue method^11^.

The rhodanese activity of full-length *Blh* or its DUF442 domain alone was determined spectrophotometrically as a measure of the iron-thiocyanate formation, using thiosulfate and cyanide as sulfur donor and acceptor, respectively^23^. The purified proteins (12 µg) were incubated at 25 °C for 5 min in 12 mM potassium phosphate buffer, pH 7.4, containing 12 mM sodium thiosulfate and 12 mM potassium cyanate. The reaction was stopped with 1.8% formalin, followed by the addition of 100 mM iron nitrate, dissolved in 14% nitric acid, and the samples were read for absorbance at 460 nm. The amount of sulfite produced in the reaction was estimated using the sulfite test strips. For estimations of the specific enzyme activity, *V*_*max*_ and *Km* of full-length *Blh*, the rhodanese reaction was performed varying the thiosulfate concentration from 0 to 100 mM.

The ST activity of full-length *Blh* or of the DUF442 domain alone was determined spectrophotometrically as a measure of sulfite production using the fuchsine method^47^. Purified proteins (12 µg) were incubated at 25 °C for 5 min in 50 mM acetate-buffer, pH 7.5, containing 10 mM sodium thiosulfate and 10 mM GSH. Alcoholic potassium hydroxide was added to samples at 0.16% final concentration. The samples were dissolved to 1:1 (V/V) in fuchsine reagent and read at 580 nm. A standard curve for sodium sulfite concentrations ranging from 0.4 µM to 2 mM was generated to estimate the amounts of sulfite in the samples. The coupled ST and SDO activities of *Blh* was determined in the oxygraph in 50 mM Tris-acetate, pH 7.4, containing 10 mM thiosulfate and 10 mM GSH, using the DUF442 and ETHE1 domains purified separately. The ST reaction started with the addition of 6 µg DUF442, and after 3 min, the ETHE1 domain (6 µg) was added to the reaction mixture. The reaction was also performed starting with the addition of the ETHE1 domain first, or using the full-length *Blh* as a control. O_2_ consumption was measured throughout the assay.

### X-ray fluorescence analysis

X-ray fluorescence measurements of the ETHE1 and BSA samples were performed at the XRF beamline of the Brazilian Synchrotron Light Laboratory (LNLS), as described previously^48^. A white beam of 0.1 mm high by 5 mm wide was used to excite the ETHE1 protein, or BSA, used as control, under total external reflection conditions of the X-ray beam for sample excitation. Samples were prepared by pipetting 5 µL of the purified proteins (~4 µg) onto a Perspex support. Samples dried at room temperature were measured for 300 s and the collected X-ray fluorescence spectra were evaluated using the PyMCA program^49^.

### Respirometry assays

High-resolution respirometry assays were performed using an Oroboros 2 K oxygraph. The *A. tumefaciens* cells were grown in YEP medium (1.0% peptone, 1.0% yeast extract, 0.5% NaCl, 1.5% agar) at 30 °C for 48 h. Bacterial colonies were suspended in AB medium^50^, supplemented with 2 mM sodium phosphate and 40 mM MES, pH 5.8, and the optical density at 600 nm immediately adjusted to 1.5. Two mL of the cell suspensions containing approximately 1.0 × 10^7^ cells were analyzed in the Oroboros equipment previously calibrated with the same medium. O_2_ consumption was recorded at 25 °C until its full depletion.

### Sulfite toxicity assay

Leaves from *Citrus sinensis* (Pineapple) plants, maintained in the greenhouse, were detached and washed with sterile water. Leaf discs were excised, embedded in water solutions of sodium sulfite or sodium sulfate at 0.5, 1.0, 5.0 and 10 mM concentrations, and placed onto filter paper wet with the same solutions inside petri dishes. The leaf discs were incubated at 25 °C for 24 to 72 h under a 12 h light/12 h dark regime, after which they were photographed.

### Plant inoculations and quantitative RT-PCR

Sweet orange Pineapple plants were infected with *X. fastidiosa* (9a5c strain) according to Almeida *et al*.^51^. Six leaves showing typical CVC symptoms, or no visible symptoms, were collected and their petioles were used for bacterial quantification. Petioles were weighed, ground in liquid nitrogen and total DNA was extracted using DNeasy Blood and Tissue kit (Qiagen). Bacterial population was determined by qPCR using a standard curve, as described previously^52^. Amplifications were performed using TaqMan PCR Master Mix (Applied Biosystems) with 10 pmol of primers CVC-1 (TGA AGA TCA TGC AAA AAA CAA) and CCSM-1 (GCGCATGCCAAGTCCATATTT), probe TAQ-CVC-(6FAM) AACCGCAGCAGAAGCCGCTCATC (TAMRA) (Applied Biosystems) and 270 ng of DNA. Reactions were performed in triplicates using an ABI PRISM 7500 Sequence Detector System (Applied Biosystems). The Ct (threshold cycle) and DNA concentration values were correlated by linear regression.

For SiR and SO quantification, total RNA was extracted from the same collected leaves using TRIzol (Thermo Fisher Scientific, USA). The quantity and quality of the RNA samples were evaluated by denatured gel electrophoresis and absorbance at 260/280 nm. cDNA was synthesized using the SuperScript III Reverse Transcriptase (Thermo Fisher Scientific), following the manufacturer’s procedure. The oligonucleotides used for the amplification of the SiR (GCCTTCGCATTCGCTCTCA/CGTACGGAACGGGAAAAGC) and SO (TCGAGGATGCCGGGACTA/ACGAGGCGGCTCTTGAGA) genes (GenBank accessions XM_006470566 and XM_006469992.2, respectively) were designed using the Applied Biosystems Primer Express 2.0 software, and the amplifications were carried out using a 7500 PCR system (Applied Biosystems, USA). The citrus actin and malate translocator genes were used as internal control for normalization^53,54^. Total RNAs from five independent leaves were used as biological replicates, and three technical replicates for each biological sample were performed. The relative gene expression levels among samples were determined using the 7000 System SDS software v2.3 (Applied Biosystems) with default parameters

### Sulfite measurements in citrus leaves

Leaf powder (250 mg fresh weight) from the same symptomatic, asymptomatic or healthy pineapple plants used for Sir and SO mRNA quantifications were incubated in 1 mL Tris-HCl pH 8.0 with 500 mM sodium sulfate solution for 10 min at 25 °C to avoid spontaneous conversion of sulfite into sulfate^55^. The suspensions were centrifuged at 18.000 × g for 5 min and the supernatants were collected and mixed with 3% KOH diluted in ethanol (1:1 v/v), as described by Leinweber and Monty^55^. Samples were centrifuged at 18.000 × g for 15 min at 4 °C and the supernatants were mixed (5:1 v/v) with fuchsine solution prepared according to Leinweber and Monty^47^. After 10 min incubation at room temperature, samples were measured for absorbance at 575 nm. The amount of sulfite in the samples was estimated using a sulfite calibration curve determined with freshly prepared sodium sulfite solutions^47^.

### Detection of H_2_S and polysulfides in elemental sulfur sources

Saturated acetonic sulfur solutions were adjusted to pH 12 with potassium hydroxide at room temperature and inspected for the yellow color production^32,33^. The polysulfide solution was prepared according to Kleinjan *et al*.^56^. Elemental sulfur (0.15 g) was dissolved into 20 mM potassium phosphate buffer, pH 8.0, containing 60 mM ammonium sulfide and the solution was incubated for 16 h at 50 °C. Polysulfide formation was evaluated by absorbance at 300–490 nm^33^.

To evaluate the presence of H_2_S in the elemental sulfur sources, bismuth citrate was added to the saturated acetonic sulfur solutions, which were inspected for a brownish-orange color.

### Incubation of *BigR* with gaseous H_2_S

Recombinant *BigR* was purified by affinity chromatography as previously described^20^. Aliquots of 0.3 mL of purified *BigR* (0.2 mg/mL) in 50 mM Tris-HCl pH 7.4, NaCl 200 mM NaCl, 10% glycerol 10% and 200 mM imidazole, were placed inside a sealed stainless-steel jar specially designed to flow gases into the sample. Nitrogen gas at 20 psi was flushed through the system for 5 min at 1 mL/min to remove O_2_. Subsequently, 5.0% H_2_S at 20 psi was flushed at 1 mL/min through the sample for up to 1 h. After the treatment, protein samples were subjected to gel-shift and mass spectrometry analysis as described below.

### Gel-shift assays

Gel-shift assays were performed as described previously^20^, except that the DNA-protein complexes were detected by ethidium bromide. Approximately 2 µg of *BigR*, purified by affinity chromatography, was incubated in binding buffer (10 mM de Tris HCl pH 7.5, 50 mM NaCl, 0.5 mM de EDTA) containing GSH, GSSH, or the acetonic sulfur solution at 100 µM, for 1 h at 4 °C. The 115 bp DNA probe (300 ng) was subsequently added to the reaction and the mixtures were further incubated at 4 °C for 30 min. *BigR* samples previously treated with H_2_S gas were also incubated 4 °C for 30 min with the DNA probe. Protein-DNA complexes were resolved on 6% polyacrylamide gels in 1X TBE buffer. After the run, gels were stained with ethidium bromide and visualized under UV light.

### Mass spectrometry analyses

Recombinant *BigR* (50 µg) incubated with gaseous H_2_S or which had been treated with either the 2.0% polysulfide solution (v/v), 125 µM GSH, 5 mM hydrogen peroxide or 5 mM dimedone in phosphate-saline (PBS) buffer for 20 min at 4 °C, were resolved on SDS-gels. *BigR* bands were removed and digested with chymotrypsin (1 µg) for 16 h at 37 °C. The samples were reduced or not with DTT and/or alkylated with iodoacetamide for mass spectrometry analysis as described by Villén and Gygi^57^. The peptides derived from the chymotrypsin-digested samples were desalted by stage-tips, dried in a vacuum concentrator and reconstituted in 50 µL of 0.1% formic acid. BigR peptides mixtures incubated with gaseous H_2_S or which had been treated with either the 2.0% polysulfide solution (v/v), 125 µM GSH, 5 mM hydrogen peroxide were separated by C18 (100 µm x 100 mm) RP-nanoUPLC (nanoAcquity, Waters) coupled with a Q-Tof Premier mass spectrometer (Waters) with nanoelectrospray source at a flow rate of 0.6 µl/min. The gradient was 2–30% acetonitrile in 0.1% formic acid over 30 min for the digested proteins. The nanoelectrospray voltage was set to 3.5 kV, a cone voltage of 30 V and the source temperature was 80 °C. The instrument was operated in the ‘top three’ mode, in which one MS spectrum is acquired followed by MS/MS of the top three most-intense peaks detected. After MS/MS fragmentation, the ion was placed on exclusion list for 60 s and for the analysis of endogenous cleavage peptides, a real-time exclusion was used. The spectra were acquired using software MassLynx v.4.1 and the raw data files were converted to a peak list format (mgf) by the software Mascot Distiller v.2.6.2.0, 2017 (Matrix Science Ldt.) and searched against a customized *Xylella* database (6,493 sequences; 2,025,062 residues) using Mascot engine v.2.3.0 (Matrix Science Ltd.), with carbamidomethylation (+57 Da) as fixed or variable modification, and oxidation of methionine (+16 Da), polysulfide or persulfide (+32, +64, +96 and +128 Da) in cysteines, considering the additional loss of one hydrogen as variable modifications. One chymotrypsin missed cleavage and a tolerance of 0.1 Da for both precursor and fragment ions were considered. For disulfide bonds analysis, a peak list (mgf) generated by Mascot Distiller v.2.6.2.0, 2017 (Matrix Science Ldt.) was analyzed in MassMatrix software^58^ to automatically search Cys-Cys disulfide bonds against database containing the *BigR* protein sequence, according to the software instructions. The parameters for disulfide bonds analysis used in MassMatrix software were carbamidomethylation (+57.021 Da), oxidation of methionine (+15.995 Da), polysulfide or persulfide (+32, +64, +96 and +128 Da) in cysteines, considering the additional loss of one hydrogen as variable modifications, non-cleavable by enzymes, two chymotrypsin missed cleavages, and a tolerance of 0.1 Da for precursor and for fragment ions.

BigR peptides mixtures treated with 5 mM dimedone in phosphate-saline (PBS) buffer were analyzed on a ETD enabled LTQ Orbitrap Velos mass spectrometer (Thermo Fisher Scientific) coupled with LC-MS/MS by an EASY-nLC system (Proxeon Biosystem). Peptides were separated by a 2–30% acetonitrile gradient in 0.1% formic acid using an analytical PicoFrit ID75 μm column (New Objective) at a flow rate of 300 nL/min over 30 min. The nanoelectrospray voltage, source temperature and instrument method were set to 2.2 kV, 275 °C and data-dependent acquisition mode. The full scan MS spectra (m/z 300-1,600) were acquired in the Orbitrap analyzer after accumulation to a target value of 1e6. The resolution in the Orbitrap system was set to r = 60,000, and the five most intense peptide ions with charge states ≥2 were sequentially isolated to a target value of 50,000 and fragmented in higher-energy collisional dissociation with normalized collision energy of 40% with the resolution in the Orbitrap system was set to r = 7,500 for MS/MS. The signal threshold for triggering an MS/MS event was set to 80,000 counts and an activation time of 0.1 ms was used. Dynamic exclusion was enabled with an exclusion size list of 400, exclusion duration of 60 s, and a repeat count of 2. The raw data files were converted to a peak list format (mgf) by the software Mascot Distiller v.2.6.2.0, 2017 (Matrix Science Ldt.) using the same parameters described above with a tolerance of 10 ppm for precursor ions and 0.02 Da for fragment ions.

### Reporter gene assays

GFP fluorescence, as a measure of operon activation, was determined in the C58 *A. tumefaciens* wild type, *blh*^*−*^ and *bigR*^*−*^ cells based on Barbosa and Benedetti^20^. Bacterial cells were grown in YEP medium for 16 h at 30 °C under agitation (200 rpm). The cells were pelleted and washed twice in 25 mM phosphate buffer, pH 7.4, containing 0.6% glucose. The cells were suspended in the same buffer and the optical density of the suspensions at 600 nm was adjusted to 1.0. The cells were incubated at 30 °C for 3 h at 100 rpm in the presence or absence of the polysulfide solution at 0.2%, 2.0% or 10% v/v. After the incubation, bacterial cells were visualized for GFP fluorescence on a Zeiss Imager A.2 fluorescence microscope.

## Electronic supplementary material

Supplementary information
