# Supplementary material for: BigR is a sulfide sensor that regulates a sulfur transferase/dioxygenase required for aerobic respiration of plant bacteria under sulfide stress
Source: Sci Rep. 2018 Feb 22;8:3508. doi: 10.1038/s41598-018-21974-x (PMC5823870; doi:10.1038/s41598-018-21974-x)
Supplement: Supplementary file 1 — Supplementary information [file 41598_2018_21974_MOESM1_ESM.pdf]

Supporting Information

BigR is a sulfide sensor that regulates a sulfur transferase/dioxygenase required for aerobic respiration of plant bacteria under sulfide stress

Nayara Patricia Vieira de Lira, Bianca Alves Pauletti, Ana Carolina Marques, Carlos Alberto Perez, Raquel Caserta; Alessandra Alves de Souza; Aníbal Eugênio Vercesi, Adriana Franco Paes Leme, Celso Eduardo Benedetti

Supplementary Tables

Supplementary Table S1. Peptides derived from chymotrypsin-digested *BigR* samples identified by mass spectrometry

Treatment	Peptide sequence	Cys modification	Cysteine	m/z (charge)	Mascot Score
**Control**	TIFCAQEKQAL	none	108	626,3194 (+2)	9
	TIFCAQEKQAL	none	108	626,3198 (+2)	11
	TIFCAQEKQAL	none	108	626,3194 (+2)	11
	TIFCAQEKQAL	none	108	626,3194 (+2)	11
	TIFCAQEKQAL	none	108	626,3204 (+2)	2
	TIFCAQEKQAL	none	108	626,3196 (+2)	5
	TIFCAQEKQAL	none	108	626,3196 (+2)	10
	CAQEKQALEHHHHHH	none	108	461,2139 (+4)	37
	CAQEKQAL	none	108	890,4352 (+1)	37
	CAQEKQAL	none	108	890,4352 (+1)	37
	VCTLVEGES	none	108	498,7376 (+2)	7
	VCTLVEGES	none	42	996,4669 (+1)	48
	VCTLVEGES	none	42	498,7381 (+1)	16
	VCTLVEGES	none	42	498,7386 (+2)	7
**Polysulfide solution**	TIFCAQEKQAL	Persulfide (SH)	108	642,3147 (+2)	26
	TIFCAQEKQAL	Persulfide (SH)	108	642,3148 (+2)	11
	TIFCAQEKQAL	Persulfide (SSH)	108	658,3016 (+2)	14
**H_2S gas (1 h)**	TIFCAQEKQAL	Persulfide (SSH)	108	658,3026 (+2)	10
	TIFCAQEKQAL	Persulfide (SSH)	108	658,3016 (+2)	9
	TIFCAQEKQAL	Persulfide (SSH)	108	658,3016 (+2)	13
	TIFCAQEKQAL	Persulfide (SSH)	108	658,3016 (+2)	9
	TIFCAQEKQAL	Persulfide (SSH)	108	658,3026 (+2)	10
	TIFCAQEKQAL	Persulfide (SSH)	108	658,3016 (+2)	13
	TIFCAQEKQAL	none	108	626,3204 (+2)	5
	TIFCAQEKQAL	none	108	626,3219 (+2)	5
**H_2S gas (30 min)**	TIFCAQEKQAL	Persulfide (SSH)	108	658,3007 (+2)	12
	TIFCAQEKQAL	Persulfide (SSH)	108	658,3012 (+2)	3
	TIFCAQEKQAL	Persulfide (SSH)	108	658,2989 (+2)	20
	TIFCAQEKQAL	Persulfide (SSH)	108	658,2995 (+2)	20
	TIFCAQEKQAL	Persulfide (SSH)	108	658,3015 (+2)	15
	TIFCAQEKQAL	none	108	626,3188 (+2)	13
	TIFCAQEKQAL	none	108	626,3190 (+2)	13
**GSH**	VCTLVEGES	Glutathionylation	42	651,2717 (+2)	25
	CAQEKQAL	Glutathionylation	108	1195,5056 (+1)	6
	TIFCAQEKQAL	Glutathionylation	108	778,8559 (+2)	0
	CAQEKQAL	Glutathionylation	108	399,1723 (+3)	4
	CAQEKQAL	Glutathionylation	108	1195,5056 (+1)	6
	CAQEKQALEHHHHHH	Glutathionylation	108	430,1856 (+5)	31
	CAQEKQALEHHHHHH	Glutathionylation	108	537,4796 (+3)	12
	CAQEKQALEHHHHHH	Glutathionylation	108	716,3045 (+4)	16
**Dimedone**	VCTLVEGES	none	42	995,4592	8
	VCTLVEGES	Sulfenic Acid	42	1133,5303	20
	CAQEKQAL	Sulfenic Acid	108	1027,4955	4
	CAQEKQALEHHHHHH	Sulfenic Acid	108	1978,8925	26
**GSH and Dimedone**	VCTLVEGES	Sulfenic Acid	42	567,7731 (+2)	18
	VCTLVEGES	Glutathionylation	42	651,2719 (+2)	12
	VCTLVEGES	Glutathionylation	42	651,2749 (+2)	12
	VCTLVEGES	Glutathionylation	42	1301,5382 (+1)	4
	CAQEKQALEHHHHHH	Sulfenic Acid	108	495,7300 (+5)	17
	CAQEKQALEHHHHHH	Glutathionylation	108	716,3047 (+3)	15
	CAQEKQALEHHHHHH	Glutathionylation	108	537,4804 (+4)	9
	CAQEKQALEHHHHHH	Glutathionylation	108	430,1863 (+5)	28
	CAQEKQAL	Glutathionylation	108	399,1725 (+2)	4
	CAQEKQAL	Glutathionylation	108	1195,5061 (+2)	3
	CAQEKQAL	Glutathionylation	108	598,2548 (+2)	2
	TIFCAQEKQAL	Glutathionylation	108	519,5761 (+3)	12
	TIFCAQEKQAL	Glutathionylation	108	778,8564 (+2)	3
	CAQEKQALEHHHHHH	Sulfenic Acid	108	495,7292 (+4)	10
	CAQEKQALEHHHHHH	Glutathionylation	108	1073,9506 (+2)	20
	CAQEKQALEHHHHHH	Glutathionylation	108	716,3037 (+3)	19
	CAQEKQALEHHHHHH	Glutathionylation	108	537,4806 (+4)	13
	CAQEKQALEHHHHHH	Glutathionylation	108	430,1863 (+5)	30

Supplementary Figures

08/08/2017 MassMatrix Result

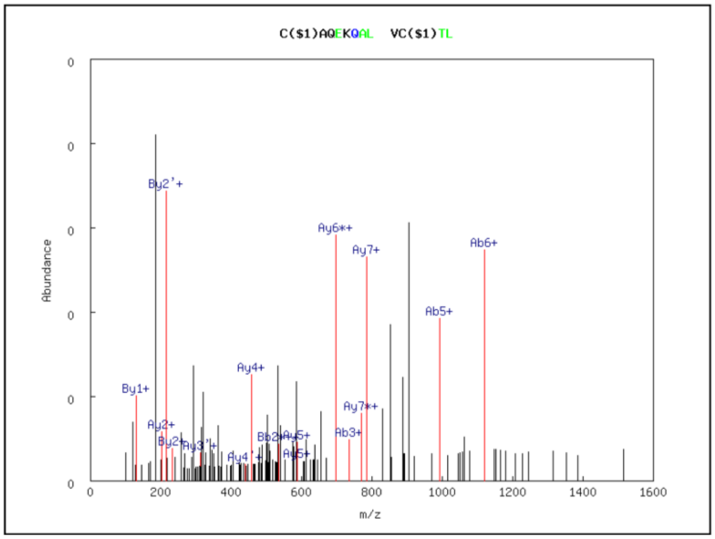


```
Index  scan# charge score   pp    pp2  pptag    m/z      MW(obs)     MW      delta   miss Unique sequence + modifications
 251    225    +2    10    11.5  16.5   3.0  661.8363  1322.6653 1322.6443 0.0210   0      √    C($1)AQEKQAL  VC($1)TL
```

chainA :

#	b'^{++}	b^{*++}	b^{++}	b'^{+}	b^{*+}	b^{+}	seq	y'^{++}	y^{*++}	y^{++}	y'^{+}	y^{*+}	y^{+}	#
1	259.61	--	268.61	518.21	--	536.22	C	652.82	653.31	661.83	1304.63	1305.62	1322.64	M
2	295.13	--	304.13	589.25	--	607.26	A	385.21	385.71	394.22	769.42	**770.40**	**787.43**	7
3	359.16	359.65	368.16	717.31	718.29	**735.32**	Q	349.70	350.19	358.70	698.38	**699.37**	716.39	6
4	423.68	424.17	432.68	846.35	847.33	864.36	E	285.67	286.16	294.67	570.32	571.31	**588.34**	5
5	487.73	488.22	496.73	974.44	975.43	**992.45**	K	221.14	221.64	230.15	**441.28**	442.27	**459.29**	4
6	551.75	552.25	560.76	1102.50	1103.49	**1120.51**	Q	157.10	157.59	166.10	**313.19**	314.17	331.20	3
7	587.27	587.77	596.28	1173.54	1174.52	1191.55	A	93.07	--	102.07	185.13	--	**203.14**	2
	--	--	--	--	--	--	L	57.55	--	66.55	114.09	--	**132.10**	1

chainB :

#	b'^{++}	b^{*++}	b^{++}	b'^{+}	b^{*+}	b^{+}	seq	y'^{++}	y^{*++}	y^{++}	y'^{+}	y^{*+}	y^{+}	#
1	--	--	50.54	--	--	100.08	V	--	--	--	--	--	--	
2	536.75	**537.24**	545.75	1072.49	1073.48	1090.50	C	603.29	603.78	612.29	1205.57	1206.55	1223.58	3
3	587.27	587.77	596.28	1173.54	1174.52	1191.55	T	108.07	--	117.08	**215.14**	--	**233.15**	2
	--	--	--	--	--	--	L	57.55	--	66.55	114.09	--	**132.10**	1

```
Spectral Info:
Scan#  tR(min)  tR(Pred)  Conf. tR  Peak Area
225    14.83    12.08      12.04%     N/A
```

```
All possible peptide matches for this spectrum
 251    225    +2    10    11.5  16.5   3.0  661.8363  1322.6653 1322.6443 0.0210   0      √    C($1)AQEKQAL  VC($1)TL
```

```
The peptide is from:
hit1 BigR
```

Supplementary Fig. S1. Identification of the *BigR* peptide containing the disulfide bond between Cys42 and Cys108 from H_2S-treated samples. Representative MS/MS spectrum showing the *b* and *y* ion series for the *BigR* peptide containing the disulfide bond involving Cys42 and Cys108, using the MassMatrix software.

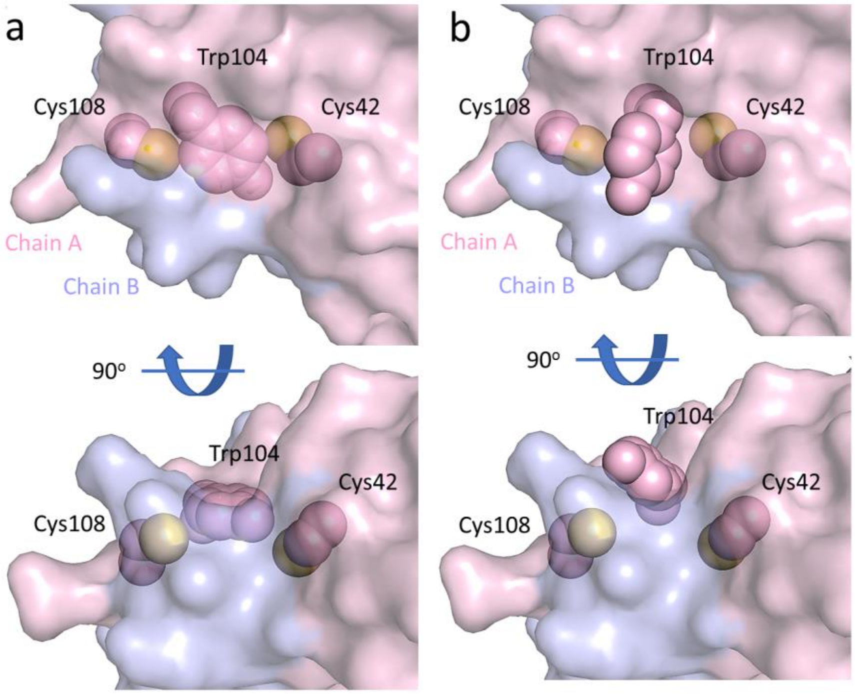

Supplementary Fig. S2. Surface representation of the Cys42 and Cys108 environment in the *BigR* dimer. **a-** In the reduced *BigR* structure (PDB code 3PQJ), Cys108 is more exposed to the solvent than Cys42, and thus more likely to react with the hydrosulfide anion to form a cysteine persulfide. The side chain of Trp104, which sits in between Cys42 and Cys108, prevents the disulfide bond formation. **b-** The side chain of Trp104 assumes a different conformation in oxidized *BigR* (PDB code 3PQK) suggesting that formation of a Cys108 persulfide might trigger the flipping of Trp104 side chain creating space for the two cysteines to interact and form the disulfide bond.
